# Clinical characteristics and prognostic impact of streptococcal colonization in critically ill patients with severe pneumonia

**DOI:** 10.3389/fcimb.2026.1647511

**Published:** 2026-01-22

**Authors:** Hang Ni, Jiaqi Zhu, Yanfang Chen, Ye Zheng, Benjia Chen, Cuicui Dong, Sheng Zhang, Yinghe Xu, Yongpo Jiang

**Affiliations:** 1 Department of Critical Care Medicine, Taizhou Hospital of Zhejiang Province, Shaoxing University, Taizhou, China; 2 Zhejiang Engineering Research Center for Intelligent Medical Imaging, Sensing and Non-invasive Rapid Testing, Taizhou, China

**Keywords:** colonization, metagenomic sequencing, pulmonary microbiome, severe pneumonia, Streptococcus

## Abstract

**Background:**

*Streptococcus* species are predominant ​commensal residents of the respiratory tract​ in healthy individuals and contribute to immune and metabolic regulation. However, the association between streptococcal colonization and clinical outcomes in patients with severe pneumonia remains undercharacterized. This study aimed to explore the clinical characteristics and the impact of streptococcal colonization on the prognosis of critically ill patients with pneumonia.

**Method:**

We conducted a multicenter, retrospective, observational cohort study of critically ill pneumonia patients admitted to 12 intensive care units (ICUs) between January 2019 and December 2023 who underwent metagenomic next-generation sequencing (mNGS). Patients were stratified into *Streptococcus*-colonized and non-colonized groups based on bronchoalveolar lavage fluid (BALF) mNGS results, conventional microbiological testing (CMT), and clinical assessments. Propensity score matching (PSM) was utilized to minimize baseline confounding variables. Using nearest-neighbor matching at a 1:2 ratio, baseline characteristics were balanced between groups post-matching. The primary endpoint was 28-day all-cause mortality.

**Results:**

A total of 1,897 patients were enrolled in this study. Among them, 21 patients under 18 years of age, 139 patients lost to follow-up within 28 days, and 4 patients with confirmed streptococcal infection were excluded. Finally, 1,733 patients met the inclusion criteria. The cohort had a mean age of 65 years, with the majority being males (1,213/1,733, 70%). Among these, 148 (8.5%) were classified as *Streptococcus*-colonized, and 1,585 (91.5%) were *Streptococcus*-colonization-negative. No significant difference in 28-day all-cause mortality was observed between the colonized and non-colonized groups (35.81% vs. 38.51%, p=0.578). Patients with *Streptococcus* colonization had a significantly shorter median length of stay (LOS) (17 days, interquartile range [IQR] 11–30) than those without colonization (22 days, IQR 12–33; P = 0.044). Similarly, their median intensive care unit (ICU) LOS (11 days, IQR 7–16) was also significantly shorter than that of non-colonized patients (14 days, IQR 8–25; P = 0.003). Multivariable Cox regression analysis further demonstrated that *Streptococcus* colonization was not an independent risk factor for 28-day mortality (HR = 1.10, 95% CI: 0.79–1.51, p=0.579).

**Conclusion:**

Our findings suggest a potential role for *Streptococcus* colonization in improving clinical outcomes in severe pneumonia. The presence or absence of *Streptococcus* colonization may influence short-term prognostic benefits in critically ill pneumonia patients. Further research is needed to clarify the clinical significance and potential mechanisms of *Streptococcus* colonization.

## 1 Introduction

Severe pneumonia continues to represent a substantial global burden of mortality.​ (Ward and Goldie, 2024). ​Current epidemiological data estimate that pneumonia necessitates ICU admission for approximately 100,000 patients worldwide each year (Mandell et al., 2007), where critical illness is associated with mortality rates reaching 20–50%​ (Lee et al., 2020). Emerging evidence reveals that the lungs, akin to the nasopharynx and gut, harbor a unique microbial ecosystem composed predominantly of bacteria, fungi, and viruses (Yu et al., 2016; Man et al., 2017). At a steady state, the lung microbiota critically maintains immune-metabolic balance, promoting pathogen clearance and suppressing maladaptive inflammation.​ (Dethlefsen et al., 2007; Mendez et al., 2019; Tomé, 2021). Notably, the diminished diversity of the respiratory microbial community has been proposed as an ecological hallmark of infection (Abreu et al., 2012). Clinically relevant perturbations characterize the lung microbiome in pulmonary disorders (Kim et al., 2017; He et al., 2022; Montassier et al., 2023; Li et al., 2024), and dysbiosis—imbalances in microbial structure and abundance—may influence disease onset, progression, and prognosis (Whiteside et al., 2021). Consequently, the respiratory microbiota ​represents a novel diagnostic biomarker enabling real-time disease surveillance​.

*Streptococcus* is the dominant colonizing flora in the lower respiratory tract (Woods et al., 2021). *Streptococcus* is taxonomically dominant within the conserved pulmonary microbiome, serving as a critical indicator of respiratory ecosystem health (Dickson et al., 2017). Among the ​primary commensals​ colonizing the human nasopharynx within hours of parturition (Bloch et al., 2024), *Streptococcus* participates in shaping complex microbial communities under both healthy and diseased conditions. ​Clinically, it sustains mucosal immune homeostasis and governs ecologically balanced colonization in the oropharyngeal microbiome (Abranches et al., 2018), serving as an initial dominant population in the human oral microbiome. This genus contributes to amino acid and nucleotide biosynthesis and is a key participant in bacterial quorum sensing (Su et al., 2023). Under specific conditions, *Streptococcus* acts as a commensal, promoting normal immune system development and inhibiting pathogen colonization (Lang et al., 2010); its metabolites (e.g., short-chain fatty acids) facilitate epithelial barrier repair and suppress excessive inflammation (Su et al., 2023). However, previous research has predominantly focused on the pathogenic potential of *Streptococcus* (Yumoto et al., 2019). Multiple studies (Erb-Downward et al., 2011; Bousbia et al., 2012) have reported no significant differences in *Streptococcus* detection rates between patient cohorts and healthy controls, reinforcing its role as a core respiratory tract colonizer. In asthma patients, lower inflammatory levels have been associated with pulmonary *Streptococcus* colonization during homeostasis (Bernasconi et al., 2016; Sharma et al., 2020). Similarly, reduced inflammation linked to *Streptococcus* has been observed in lung transplant recipients (Bernasconi et al., 2016), whereas in idiopathic pulmonary fibrosis (IPF) patients, *Streptococcus* colonization correlates with faster disease progression (O’Dwyer et al., 2019; Invernizzi et al., 2020); *Streptococcus* biomass accumulation remodels the lung tumor immune microenvironment via PI3K/ERK-dependent epithelial reprogramming, potentiating myeloid cell infiltration (Gustafson et al., 2010). These findings underscore the significant yet context-dependent role of *Streptococcus* colonization across respiratory pathologies. A microbiome investigation of severe pneumonia stratified patients into deceased and survivor cohorts according to 28-day survival outcomes. The survivor group exhibited *Streptococcus* as a core genus within the respiratory microbiota. Nevertheless, this study did not elucidate the mechanistic association between *Streptococcus* colonization and severe pneumonia prognosis (Huang et al., 2024).

Therefore, this study aimed to investigate the association between *Streptococcus* colonization and clinical outcomes in patients with severe pneumonia using metagenomic next-generation sequencing (mNGS).​ By analyzing the microbial composition data obtained from mNGS and the clinical data, we attempt to describe the clinical characteristics and prognostic analysis of *Streptococcus* colonization.

## 2 Materials and methods

### 2.1 Clinical data

This multicenter, retrospective, observational cohort study was conducted across 12 intensive care units (ICUs) in China between January 2019 and December 2023. The study has been approved by the ethics committees of all participating hospitals (Approval No.:K20230510). All patients with severe pneumonia who required bronchoalveolar lavage fluid (BALF) metagenomic next-generation sequencing (mNGS) were included. The mNGS laboratory workflow received dual accreditation under CAP (College of American Pathologists) standards and China NHC EQA (National Health Commission External Quality Assessment) protocols.​ Exclusion criteria were as follows: 1. Aged <18 years; 2. No mNGS testing performed within 28 days of ICU admission; 3. No conventional microbiological testing (CMT) samples were collected within 28 days of ICU admission; 4. Lost to follow-up or withdrew treatment within 28 days of ICU admission; 5. Patients with confirmed *Streptococcus* infections.

### 2.2 Colonization vs. pathogenicity

Streptococcus infection was defined as: (1) positivity for *Streptococcus* by conventional microbiological testing (CMT) and (2) clinical symptoms consistent with streptococcal infection. In this study, *Streptococcus* colonization was defined as: (1) detection of *Streptococcus* via metagenomic sequencing (mNGS) and negative CMT cultures, and (2) clinical assessment by two independent clinicians with titles of associate chief physician or higher, specializing in infectious diseases. Discrepancies in judgments were resolved by a third senior chief physician to ensure diagnostic accuracy.

### 2.3 Data collection

Demographic data (sex, age), medical history (community-acquired pneumonia, immune status, diabetes, myocardial infarction, chronic pulmonary/liver/kidney diseases, solid/hematologic malignancies, connective tissue disorders, transplantation history), clinical scores (worst Sequential Organ Failure Assessment [SOFA] score within the first 24 hours of ICU admission and within 24 hours preceding mNGS testing), mNGS results, and CMT findings were extracted from electronic medical records. Clinicians identified causative pathogens based on clinical data, excluding patients with confirmed *Streptococcus* infections. Participants were stratified into *Streptococcus*-colonized and colonization-negative groups using mNGS results. Propensity score matching (PSM, 1:2 ratio) was applied to balance baseline characteristics (age, sex, SOFA scores at ICU admission and mNGS testing, community-acquired pneumonia, immune status, comorbidities). Post-matching clinical outcomes were compared between groups, including the primary endpoint (28-day mortality) and secondary endpoints (ICU length of stay, total hospitalization duration).

### 2.3 Statistical methods

Continuous variables were analyzed using Student’s t-tests, while categorical variables were assessed via chi-square or Fisher’s exact tests. Propensity score matching (PSM) was performed using the “MatchIt” package in R software, employing a 1:2 nearest-neighbor matching algorithm with a caliper width of 0.02 to balance baseline characteristics (standardized mean difference [SMD] >0.2) between the *Streptococcus*-colonized and colonization-negative groups. Kaplan-Meier survival curves were generated to compare mortality differences between cohorts. In the matched cohort, multivariable Cox proportional hazards models were applied to evaluate the impact of *Streptococcus* colonization on 28-day mortality. All analyses were conducted using R software (v4.2.3), with statistical significance defined as p < 0.05 (two-tailed). Sensitivity analyses included 28-day mortality post-mNGS testing and subgroup assessments.

## 3 Result

### 3.1 Comparison of clinical baseline characteristics between the two groups of patients before and after matching

**Figure 1:**
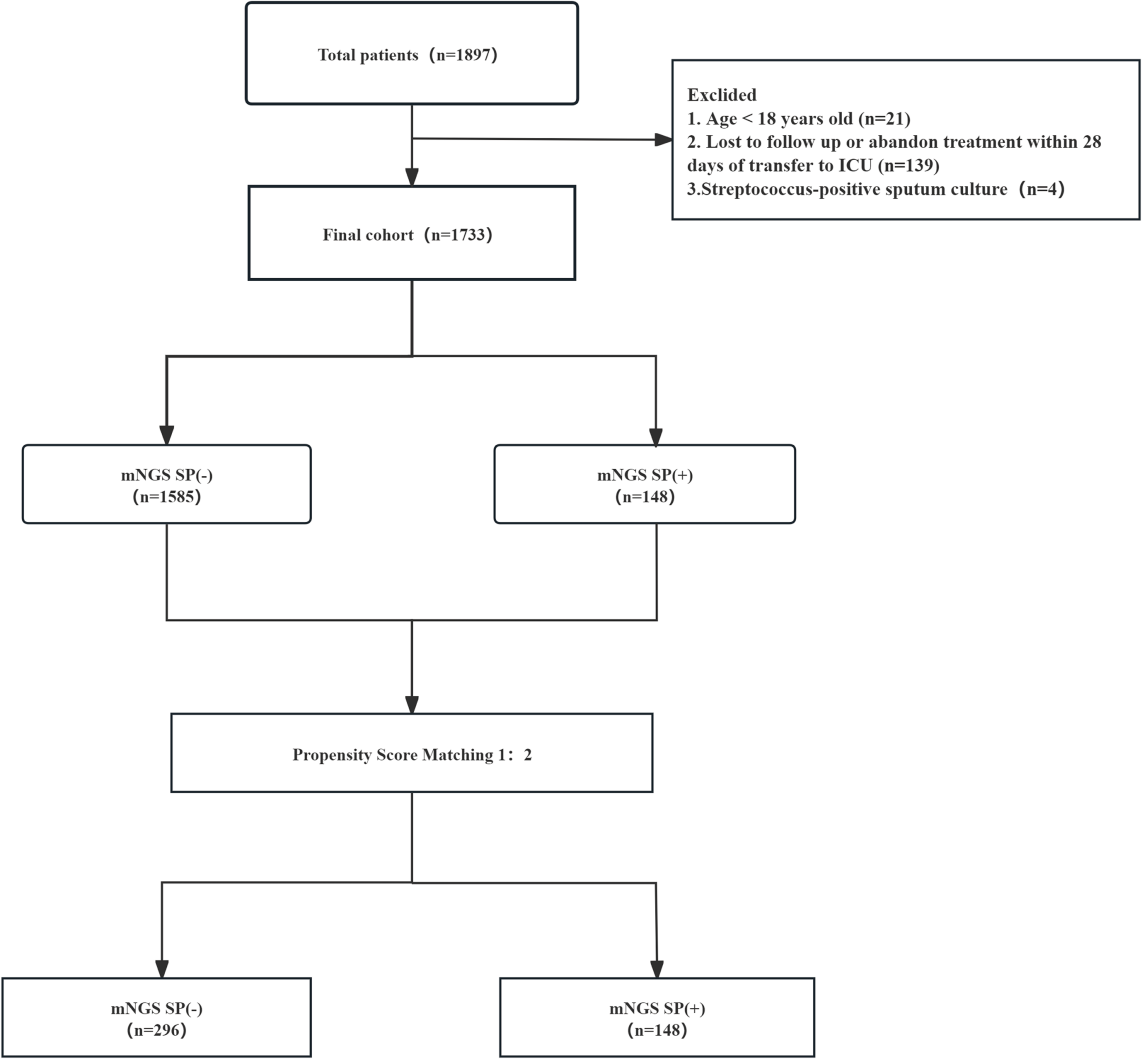
Flow diagram of this study.

Among 1,897 patients initially identified with severe pneumonia, 1,733 were eligible for final inclusion after screening (**Figure 1**). Among these, 148 patients (8.54%) were classified into the *Streptococcus*-colonized group, 1,586 (91.46%) into the colonization-negative group, and 4 patients with confirmed streptococcal infection were excluded(**Supplementary Table 2**). Baseline demographic and clinical characteristics before and after matching are detailed in **Table 1**.

**Table 1:** Characteristics of patients in the original cohort and the propensity score-matched cohort.

Variables	Original cohort				Matched cohort			
	SP(-) (n=1585)	SP(+) (n= 148)	p	SMD	SP(-) (n=296)	SP(+) (n= 148)	p	SMD
Age, mean (SD)	64 ± 16	65 ± 16	0.445	0.068	63 ± 16	65 ± 16	0.158	0.146
Male, n (%)	1103 (69.59)	110 (74.32)	0.229	0.108	205 (69.26)	110 (74.32)	0.268	0.116
Comorbidities, n (%)								
Diabetes mellitus, n (%)	392 (24.73)	34 (22.97)	0.635	-0.042	77 (26.01)	34 (22.97)	0.485	-0.072
Myocardial infarction, n (%)	90 (5.68)	12 (8.11)	0.230	0.089	24 (8.11)	12 (8.11)	1.000	0.000
Chronic pulmonary disease, n (%)	328 (20.69)	21 (14.19)	0.059	-0.186	53 (17.91)	21 (14.19)	0.322	-0.107
Liver disease, n (%)	106 (6.69)	12 (8.11)	0.512	0.052	18 (6.08)	12 (8.11)	0.422	0.074
Renal disease, n (%)	204 (12.87)	12 (8.11)	0.093	-0.174	39 (13.18)	12 (8.11)	0.114	-0.186
Solid tumor, n (%)	244 (15.39)	16 (10.81)	0.135	-0.148	39 (13.18)	16 (10.81)	0.476	-0.076
Hematological malignancy, n (%)	89 (5.62)	2 (1.35)	0.026	-0.369	4 (1.35)	2 (1.35)	1.000	0.000
CTD, n (%)	72 (4.54)	2 (1.35)	0.066	-0.276	4 (1.35)	2 (1.35)	1.000	0.000
Transplantation, n (%)	84 (5.30)	3 (2.03)	0.081	-0.232	6 (2.03)	3 (2.03)	1.000	0.000
CBD, n (%)	248 (15.65)	31 (20.95)	0.093	0.130	41 (13.85)	31 (20.95)	0.056	0.174
CAP, n (%)	945 (59.62)	102 (68.92)	0.027	0.201	204 (68.92)	102 (68.92)	1.000	0.000
Immunosuppressive status, n (%)	411 (25.93)	18 (12.16)	<.001	-0.421	36 (12.16)	18 (12.16)	1.000	0.000
SOFA score at ICU admission, mean (SD)	7.07 ± 3.66	6.80 ± 3.99	0.393	-0.068	6.88 ± 3.59	6.80 ± 3.99	0.836	-0.019
SOFA score at NGS time, mean (SD)	7.82 ± 3.93	7.50 ± 3.89	0.350	-0.081	7.59 ± 3.85	7.50 ± 3.89	0.821	-0.023
Mode Of Ventilator, n (%)			0.212				0.871	
IMV	1384 (87.32)	123 (83.11)		-0.112	250 (84.46)	123 (83.11)		-0.036
NIV	48 (3.03)	4 (2.70)		-0.020	9 (3.04)	4 (2.70)		-0.021
Other	153 (9.65)	21 (14.19)		0.130	37 (12.50)	21 (14.19)		0.048

ICU, intensive care unit; SD, standard deviation; MI, myocardial infarction; CTD, connective tissue disease; CAP, community-acquired pneumonia; SOFA, sequential organ failure assessment; NGS, next-generation sequencing; LOS, length of stay.

The *Streptococcus*-colonized group was slightly older than the colonization-negative group (65 ± 16 vs. 64 ± 16 years, p=0.445; SMD = 0.068), with no significant age disparity in the original cohort. Males comprised 74.32% of the colonized group versus 69.59% of the non-colonized group (p=0.229; SMD = 0.108), indicating balanced sex distribution. The overall prevalence of community-acquired pneumonia (CAP) was 60.42% (n=1,047). CAP was significantly more frequent in the *Streptococcus*-colonized group (68.92% vs. 59.62%, p=0.027; SMD = 0.201). Immunocompromised status was less common among colonized patients (12.16% vs. 25.93%, p<0.001; SMD=-0.421). For comorbidities, chronic pulmonary disease prevalence trended lower in the colonized group (14.19% vs. 20.69%, p=0.059; SMD=-0.186), while hematologic malignancies were significantly less frequent (1.35% vs. 5.62%, p=0.026; SMD=-0.369). No significant differences were observed in diabetes (p=0.675), myocardial infarction (p=0.145), liver diseases (p=0.555), or solid tumors (p=0.111). The *Streptococcus*-colonized group had numerically lower SOFA scores at ICU admission (6.80 ± 3.99 vs. 7.07 ± 3.66) and at mNGS testing (7.50 ± 3.89 vs. 7.82 ± 3.93), with no statistically significant differences between groups (p > 0.05).To minimize confounding, nearest-neighbor matching (1:2 ratio, caliper width=0.02) was applied to variables with SMD >0.2. Post-matching, baseline characteristics—including sex, age, immune status, comorbidities, and SOFA scores—were well-balanced between groups. Absolute SMD values for CAP (p=1.000), immunocompromised status (p=1.000), hematologic malignancies (p=1.000), connective tissue disorders (p=1.000), myocardial infarction (p=1.000), and transplantation history (p=1.000) were <0.1, indicating negligible residual differences. Age (p=0.146), male sex (p=0.116), chronic pulmonary disease (p=0.322), renal disease (p=0.114), and congestive heart failure (p=0.056) showed minimal residual imbalances (absolute SMD <0.2), none reaching statistical significance.

### 3.2 ​Comparison of clinical outcomes between groups before and after matching

We further compared 28-day mortality, total hospitalization duration, ICU length of stay, and mechanical ventilation time between the *Streptococcus*-colonized and colonization-negative groups before and after matching. In the original cohort, the primary outcome of 28-day mortality was numerically lower in the *Streptococcus*-colonized group (38.51% vs. 41.58%), though this difference lacked statistical significance (p=0.469). Patients with *Streptococcus* colonization had a significantly shorter median length of stay (LOS) (17 days, interquartile range [IQR] 11–30) than non-colonized patients (22 days, IQR 12–37; p=0.009). Similarly, their median intensive care unit (ICU) LOS (11 days, IQR 7–16) was also significantly shorter than that of non-colonized patients (13 days, IQR 8–24; p=0.003) (**Supplementary Table S1**). In the matched cohort, 163 patients (57 colonized vs. 106 non-colonized) died within 28 days, with no significant intergroup difference in mortality (p=0.578). For secondary outcomes, The SP(+) group showed significantly shorter median LOS (17 days, IQR 11–30) vs. SP(−) (22 days, IQR 12–33; p=0.044) and ICU LOS (11 days, IQR 7–16) vs. SP(−) (14 days, IQR 8–25; p=0.003) (**Table 2**).

**Table 2:** Primary and secondary outcomes in the propensity score-matched cohort.

Variables	Total (n = 444)	SP(-) (n = 296)	SP(+) (n = 148)	*P*
Primary outcomes				
Death 28day, n(%)				0.578
NO	281 (63.29)	190 (64.19)	91 (61.49)	
YES	163 (36.71)	106 (35.81)	57 (38.51)	
Secondary outcomes				
Los, M (Q_1_, Q_3_)	20 (11, 33)	22 (12, 33)	17 (11, 30)	0.044
Iculos, M (Q_1_, Q_3_)	13 (7, 23)	14 (8, 25)	11 (7, 16)	0.003
Ventilation Time With 28 Icu Days, M (Q_1_, Q_3_)	8 (3, 15)	8 (3, 16)	7 (3, 13)	0.203

ICU, intensive care unit; LOS, length of stay.

### 3.3 Bacterial distribution in *Streptococcus*-colonized patients

**Figure 2:**
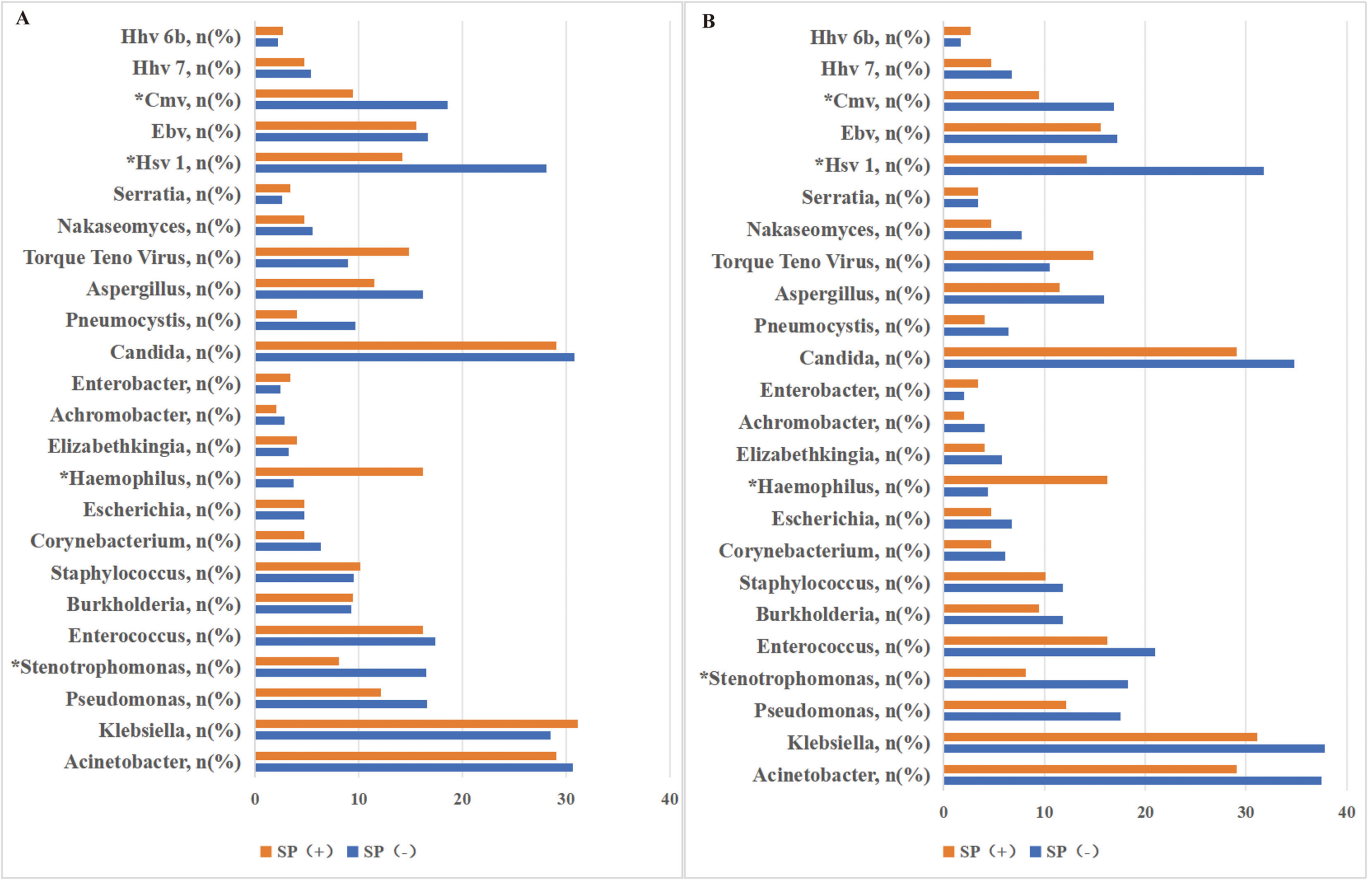
Streptococcus colonization was positive, and *Streptococcus* was the dominant microbial pathogen detected by mNGS in BALF, including bacteria, viruses, and fungi. *p<0.05 was considered significant.

We further analyzed the detection rates of other bacterial species across groups. *Stenotrophomonas* was significantly more prevalent in the *Streptococcus*-colonization-negative group compared to the colonized group (21.6% vs. 8.1%; p< 0.001), whereas *Haemophilus* species demonstrated higher detection rates in the *Streptococcus*-colonized group (16.2% vs. 6.1%; p< 0.001). Among other bacteria, the highest detection rates were observed for *Acinetobacter* (37.8% vs. 29.1%; p= 0.067) and *Klebsiella* (38.2% vs. 31.1%; p= 0.142), though intergroup differences were non-significant. The lowest detection rates were noted for *Achromobacter* (4.4% vs. 2.0%; p= 0.208) and *Enterobacter* (2.0% vs. 3.4%; p= 0.589). No statistically significant differences in bacterial distribution were observed between groups (**Figure 2**).

In the fungal detection, the observed fungal species included *Candida, Pneumocystis, Aspergillus, Nakaseomyces*, and *Serratia*, and the fungal detection rate was significantly higher in the *Streptococcus*-free colonization cohort. Specifically: Analysis of the original cohort: The detection rate of *Pneumocystis* in the *Streptococcus* colonization group (4.05%) was significantly lower than that in the non-colonization group (9.65%, p= 0.024). After matching, the detection rate of *Pneumocystis* in the *Streptococcus* colonization group (4.05%) was still lower than that in the non-colonization group (6.42%), but the difference was not statistically significant (p> 0.05) (**Figure 2**).

Viral detection results revealed that the predominant viral types identified included Torque Teno virus, herpes simplex virus type 1 (HSV-1), Epstein-Barr virus (EBV), cytomegalovirus (CMV), human herpesvirus 7 (HHV-7), and human herpesvirus 6B (HHV-6B). Specifically, HSV-1: The detection rate of HSV-1 was significantly higher in the *Streptococcus*-free colonization group (31.76%) compared to the *Streptococcus* colonization group (14.19%, p< 0.001). CMV: Similarly, CMV exhibited a significantly higher detection rate in the *Streptococcus*-free group (16.89%) than in the colonization group (9.46%, p=0.006). While *Streptococcus* colonization showed significant associations with clinical outcomes (**Table 2**), no statistically significant differences were observed in the distribution of remaining viruses between the two groups (p> 0.05 for all) (**Figure 2**).

### 3.4 Kaplan-meier analysis and multivariable Cox regression

**Figure 3:**
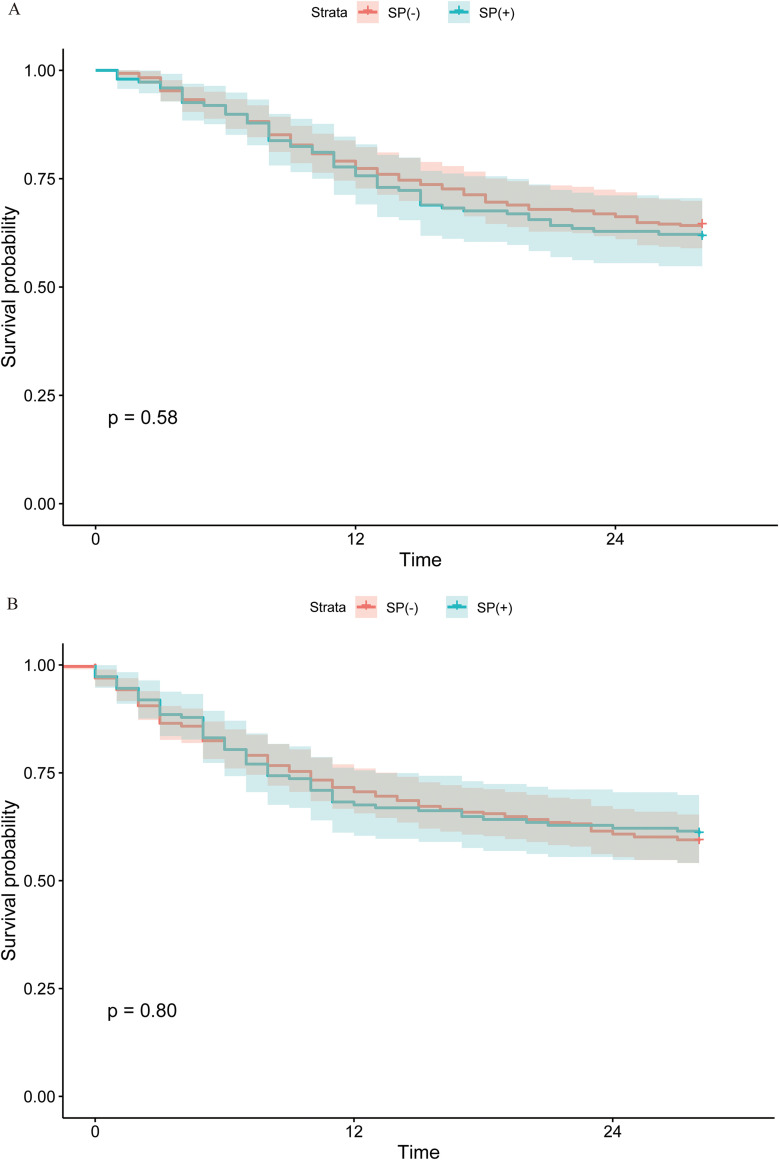
Kaplan-Meier curves and immortal time curves of the propensity score-matched cohort. **(A)** Curves for 28-day cumulative survival rates. Log-rank testing indicated no significant difference in 28-day cumulative survival between the two groups. **(B)** Curves for 28-day all-cause mortality post-metagenomic next-generation sequencing (mNGS) testing. Log-rank testing revealed no statistically significant intergroup difference in 28-day all-cause mortality.

Univariate Kaplan-Meier survival analysis was performed to compare 28-day cumulative survival rates between *Streptococcus*-colonized and non-colonized groups in the matched cohort. Log-rank testing revealed no survival benefit associated with *Streptococcus* colonization (HR = 1.097, 95% CI: 0.795–1.514, p=0.575) (**Figure 3A**). Similarly, comparison of 28-day all-cause mortality post-mNGS testing demonstrated no statistically significant intergroup difference (HR = 0.962, 95% CI: 0.704–1.316, p=0.805) (**Figure 3B**). Multivariable Cox proportional hazards models were constructed to assess the impact of *Streptococcus* colonization on 28-day mortality in the matched cohort (**Table 3**). In the crude model, *Streptococcus* colonization was not an independent risk factor for mortality (HR = 1.10, 95% CI: 0.79–1.51, p=0.579). Model 2, adjusted for age and sex, yielded comparable results (HR = 1.07, 95% CI: 0.78–1.48, p=0.664), indicating minimal confounding by these variables. Model 3, further adjusted for diabetes, myocardial infarction (MI), liver disease, chronic kidney disease (CKD), hematologic malignancies (HM), and transplantation history, similarly showed no significant association (HR = 1.10, 95% CI: 0.79–1.52, p=0.569). Model 4, incorporating additional adjustments for *Stenotrophomonas* and *Haemophilus* sp*ecies*, confirmed the absence of a significant relationship (HR = 1.20, 95% CI: 0.86–1.68, p=0.291).

**Table 3:** Multi-model Cox, the proportional hazards model.

Variables	Model 1		Model 2		Model 3		Model 4	
	HR (95%CI)	*P*	HR (95%CI)	*P*	HR (95%CI)	*P*	HR (95%CI)	*P*
SP(-)	1.00 (Reference)		1.00 (Reference)		1.00 (Reference)		1.00 (Reference)	
SP(+)	1.10 (0.79 ~ 1.51)	0.579	1.07 (0.78 ~ 1.48)	0.664	1.10 (0.79 ~ 1.52)	0.569	1.20 (0.86 ~ 1.68)	0.291

HR, Hazard Ratio; CI, Confidence Interval.

Model 1: Crude.

Model 2: Adjust: sex, age.

Model 3: Adjust: sex, diabetes, MI, liver_disease, CKD, HM, age.

Model 4: Adjust: sex, diabetes, MI, liver_disease, CKD, HM, Stenotrophomonas, Haemophilus, age.

### 3.5 Subgroup analysis

**Figure 4:**
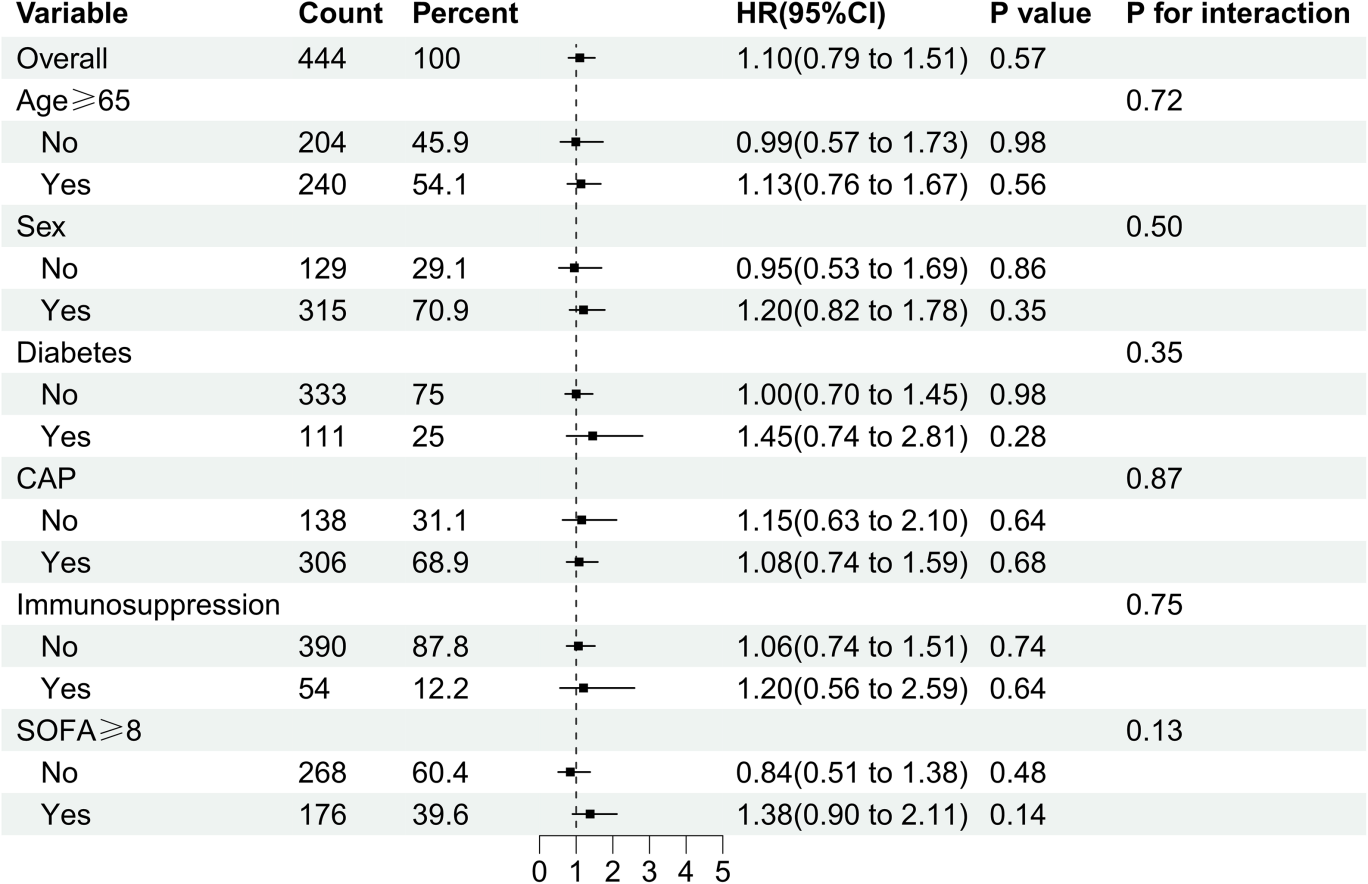
Forest plot showing subgroup-specific hazard ratios (HR) and 95% confidence intervals (CI) for the association between Streptococcus colonization status (SP+: colonized; SP-: non-colonized) and clinical outcomes in the propensity score-matched cohort (n=444).

Subgroup analyses revealed an adjusted hazard ratio (HR) of 1.10 (95% CI: 0.79–1.51; P = 0.579) for the primary endpoint in the overall population, indicating no statistically significant intergroup difference. Age-stratified analysis (cutoff: 65 years)​​ revealed: ≥65 years group: adjusted HR 1.13 (95% CI: 0.76–1.67; P = 0.56); <65 years group: adjusted HR 0.99 (95% CI: 0.57–1.73; P = 0.98) Interaction P = 0.72, indicating no significant effect modification by age on *Streptococcus* colonization outcomes. SOFA score stratification at admission (cutoff: 8 points)​​: SOFA ≥8: adjusted HR 1.38 (95% CI: 0.90–2.11; P = 0.14); SOFA <8: adjusted HR 0.84 (95% CI: 0.51–1.38; P = 0.48) Interaction P = 0.13, suggesting no significant effect modification by SOFA score on the *Streptococcus* colonization-outcome association. Stratified Analysis by Community-Acquired Pneumonia (CAP) Status​: CAP patients: adjusted HR 1.08 (95% CI: 0.74–1.59; P = 0.68) Non-CAP patients: adjusted HR 1.15 (95% CI: 0.63–2.10; P = 0.64) Interaction P = 0.87, demonstrating no significant effect modification by CAP status. Additional Subgroup Analyses​ Sex, diabetes mellitus, and immunosuppression status subgroups consistently showed non-significant associations: All adjusted HR P-values > 0.05; All interaction P-values > 0.05. This confirms the stability of the *Streptococcus* colonization-outcome association across diverse clinical profiles (**Figure 4**).

## 4 Discussion

​To our knowledge, this study is the first to uniquely focus on elucidating the association between *Streptococcus* colonization and prognosis in severe pneumonia patients. We collected respiratory tract specimens from patients and employed both metagenomic next-generation sequencing (mNGS) and culture-based microbial testing (CMT) to determine *Streptococcus* colonization status in severe pneumonia cases, with final clinical adjudication performed by physicians. The results suggest that *Streptococcus* colonization may exert potential beneficial effects on improving prognosis in critically ill patients.

Our study observed a 28-day mortality rate of 41.32% post-admission. ​Systematic reviews report 20-50% mortality among ICU-admitted severe pneumonia patients, consistent with multinational registry data (Lee et al., 2020), which aligns with our findings. However, selection bias may lead to underestimation of true mortality rates, as the cohort excluded critically ill patients who did not meet the criteria for metagenomic next-generation sequencing (mNGS) sampling.​ Additionally, no statistically significant difference in 28-day mortality was observed between the two patient groups (p > 0.05). Based solely on this pivotal clinical endpoint, *Streptococcus* colonization status did not exert a significant impact on short-term survival outcomes. Nevertheless, upon further analysis of secondary clinical outcomes, we identified significantly shorter total hospital stays and ICU lengths of stay (LOS) in the *Streptococcus*-colonized group compared to the non-colonized group (p < 0.05). These findings preliminarily suggest that *Streptococcus* colonization may exert a beneficial effect on reducing total hospital stay and ICU length of stay (LOS). Notably, the results remained highly consistent before and after propensity score matching (p < 0.05), further reinforcing the reliability and robustness of this observation. Importantly, a previous study investigating microbial characteristics in survivors versus non-survivors of severe pneumonia identified *Streptococcus* as a core genus in the survivor group, with positive correlations observed across its subspecies. (Huang et al., 2024) This aligns with our current findings in severe pneumonia patients, implying that *Streptococcus* may play a multifaceted role in disease progression and influence short-term prognosis. Subsequent analysis of bacterial detection profiles before/after matching and interventions (**Figure 4**), revealed comparable prevalence of *Acinetobacter* and *Klebsiella* between *Streptococcus*-colonized and non-colonized groups. However, *Stenotrophomonas* detection was significantly lower (8.1% vs. 21.6%), and *Haemophilus* prevalence was higher (16.2% vs. 6.0%) in the colonized group. However, *Stenotrophomonas* detection was significantly lower (8.1% vs. 21.6%) and *Haemophilus* prevalence was higher (16.2% vs. 6%) in the colonized group. This microbial shift may arise from interspecies interactions: prior studies have documented *Haemophilus*-Streptococcus co-colonization (Lewnard et al., 2019), while research on pediatric Mycoplasma pneumoniae pneumonia demonstrated that increased *Streptococcus* and *Prevotella* abundance displaces *Stenotrophomonas*, with their metabolic production of lactate promoting *Veillonella* growth and anti-inflammatory short-chain fatty acids (SCFAs) (Wei et al., 2024). Analogous mechanisms might operate in severe pneumonia. However, the lack of longitudinal microbial dynamics precluded further mechanistic exploration. A key finding of the present study was that the detection rates of herpes simplex virus type 1 (HSV-1, 31.76% vs 14.19%) and cytomegalovirus (CMV, 16.89% vs 9.46%) were significantly higher in the Streptococcus-negative group (SP-) than in the Streptococcus-positive group (SP+). This observation suggested that Streptococcus colonization may be associated with the risk of respiratory herpesvirus infection. The above finding is consistent with the well-established consensus that interactions exist between the respiratory microbiota and viral pathogens, and also possesses distinct clinical implications. Our previous work has verified a prominent synergistic effect between HSV-1 and CMV, with their co-detection being closely correlated with an elevated 28-day mortality rate among critically ill patients (Liu et al., 2025). Moreover, accumulating evidence has demonstrated that respiratory commensal bacteria are capable of modulating host susceptibility to viral infections (Kim et al., 2019). Nevertheless, as a core commensal bacterium of the respiratory tract, the direct interaction between Streptococcus, HSV-1, and CMV has not been clearly elucidated in existing literature, which thus merits further in-depth investigations.

Previous studies have established colony-forming units (CFUs) as a critical metric for determining microbial pathogenicity (Bousbia et al., 2012). For defining lower respiratory tract colonization, a bronchoalveolar lavage (BAL) culture threshold of ≥10³ CFU·mL^−1^ is widely adopted as evidence of bacterial colonization (Cabello et al., 1997), though some studies employ higher thresholds to refine pathogenicity assessments. Clinically, the principle “higher bacterial loads correlate with stronger infection relevance” guides pathogen identification (Chang et al., 2018), and positive cultures from lower respiratory specimens are typically classified as pathogens (Kitsios et al., 2018). However, metagenomic next-generation sequencing (mNGS)-based diagnosis represents an emerging field lacking standardized thresholds analogous to CFU-based criteria for distinguishing colonization from infection (Metlay et al., 2019). In practice, mNGS demonstrates a limited capacity for pathogen quantification, focusing primarily on presence/absence detection. Consequently, mNGS is rationally considered a qualitative assay (Diao et al., 2023), as it cannot discriminate between infection and colonization (Diao et al., 2023). Crucially, neither culture results nor microbial DNA sequencing alone suffices to delineate colonization versus infection; this requires comprehensive integration of clinical symptoms, radiological features, and systemic/local host responses. Therefore, this study utilized clinical microbiological testing (CMT) as the gold standard, wherein clinicians integrated multi-dimensional clinical parameters to adjudicate *Streptococcus* colonization status. Although this approach may introduce inter-rater variability, it aligns with real-world clinical decision-making workflows, thereby enhancing the clinical translatability of our findings.

The causal relationship between *Streptococcus* colonization and prognosis of severe pneumonia, as well as its colonization dynamics, remains under investigated, despite accumulating evidence highlighting its pathogenic significance in respiratory disorders (Preston et al., 2011; Segal et al., 2016; Invernizzi et al., 2020; Narciso et al., 2025). Notably, *Streptococcus* exerts heterogeneous functional roles in the lower respiratory tract across disease states—for instance, promoting pulmonary carcinogenesis in cancer patients and accelerating progression in idiopathic pulmonary fibrosis—precluding simplistic pathogenic classification (Tsay et al., 2018). Although the specific mechanisms underlying *Streptococcus* colonization-related effects remain unclear, prior studies have identified potential regulatory pathways: *Streptococcus* may ameliorate microbial dysbiosis via quorum-sensing-mediated oral biofilm stabilization (Su et al., 2023), reduce secondary infection risk through bacteriocin production (Wescombe et al., 2009; Khan et al., 2019), and modulate host immune responses (Preston et al., 2011), with preclinical evidence supporting its efficacy in alleviating recurrent pharyngitis and pathogen colonization (Deasy et al., 2015; Andaloro et al., 2019)Nevertheless, research on *Streptococcus*-supplemented probiotic interventions for respiratory diseases is still exploratory. Whether probiotics confer benefits in respiratory diseases by directly targeting lung parenchyma or restoring upper airway/intestinal microbiota homeostasis, along with their specific regulatory pathways, awaits further verification.

*Streptococcus* persists as a core genus in survivors of severe pneumonia. However, critical questions remain unresolved: Whether *Streptococcus* colonization synergizes with established pathogens to exacerbate pulmonary infection severity, whether it suppresses other pathogens through competitive inhibition, and whether it merely exists as a commensal without substantial clinical impact. Elucidating the ecological role of *Streptococcus* in this complex microenvironment holds significant implications for deciphering pulmonary infection pathogenesis, developing targeted therapeutic strategies, and improving patient prognosis.

Our study has several important limitations requiring careful consideration: Antimicrobial therapy, the cornerstone of severe pneumonia management (Zhao et al., 2023), was not systematically tracked.

Empirical broad-spectrum antibiotics are typically initiated within hours of ICU admission, profoundly altering lung microbiome composition within days – previous evidence shows sustained diversity reduction after just 72 hours of treatment (Torres et al., 2016). This missing pharmacological metadata may introduce potential confounding factors, as antibiotic-induced dysbiosis could independently affect outcomes. Single-timepoint mNGS profiling​ limited our capacity to characterize dynamic evolutionary profiles of *Streptococcus* and other genera at taxonomic resolution. The temporal dynamics of microbiome restructuring were not systematically analyzed. However, the observed depletion of *Streptococcus* (a core microbiome component) aligns with previous reports linking microbial community destabilization to adverse outcomes (Huang et al., 2024), reinforcing the pathological significance of pulmonary microbiome imbalance. Notably, although inter-group mortality showed no statistical difference, the significant disparities in total hospital stays and ICU length of stay (LOS) suggest pulmonary microbiome modulation could emerge as a therapeutic target for improving prognosis. Promising preclinical evidence exists: murine models demonstrate airway-targeted microbial modulation suppresses allergic immunity in asthma (Preston et al., 2011), while bacterial oral/nasal sprays reduce pharyngitis recurrence and pathogen colonization in clinical trials (Deasy et al., 2015; Andaloro et al., 2019). Nevertheless, key questions remain unresolved: Whether probiotic supplementation or microbial consortia transplantation can restore respiratory homeostasis; Whether direct lung parenchymal delivery outperforms upper airway/gut microbiome modulation. These knowledge gaps underscore the need for mechanistic studies exploring microbial therapeutics in critical respiratory illnesses.

## Data Availability

The datasets presented in this article are not readily available because The data can be obtained from the corresponding author YJ upon reasonable request. Requests to access the datasets should be directed to jyongpo8@163.com.
