# Supplementary material for: Clinical characteristics and prognostic impact of streptococcal colonization in critically ill patients with severe pneumonia
Source: Front Cell Infect Microbiol. 2026 Jan 22;16:1647511. doi: 10.3389/fcimb.2026.1647511 (PMC12872922; doi:10.3389/fcimb.2026.1647511)
Supplement: Supplementary file 1 [file Table1.docx]

Supplementary Table 1. Primary and Secondary Outcomes.

| Variables | Total (n = 1733) | SP（-）  (n = 1585) | SP（+）  (n = 148) | *P* |
| --- | --- | --- | --- | --- |
|  |  |  |  |  |
| **Primary Outcomes** |  |  |  |  |
| Death 28day, n(%) |  |  |  | 0.469 |
| NO | 1017 (58.68) | 926 (58.42) | 91 (61.49) |  |
| YES | 716 (41.32) | 659 (41.58) | 57 (38.51) |  |
| **Secondary Outcomes** |  |  |  |  |
| Los, M (Q₁, Q₃) | 21 (12, 36) | 22.00 (12, 37) | 17.00 (11, 30) | 0.009 |
| Iculos, M (Q₁, Q₃) | 13 (7, 23) | 13.00 (8, 24) | 11.00 (7, 16) | 0.003 |
| Ventilation Time With 28 Icu Days, M (Q₁, Q₃) | 8 (3, 16) | 8.00 (3, 16) | 7.00 (3, 13) | 0.090 |

ICU, intensive care unit; LOS, length of stay
