# Supplementary material for: Clinical characteristics and prognostic impact of streptococcal colonization in critically ill patients with severe pneumonia
Source: Front Cell Infect Microbiol. 2026 Jan 22;16:1647511. doi: 10.3389/fcimb.2026.1647511 (PMC12872922; doi:10.3389/fcimb.2026.1647511)
Supplement: Supplementary file 2 [file Table2.docx]

Supplementary Table 2. Patients with identified streptococcal pathogenesis.

| Patient ID | Age | Sex | Pathogenic bacteria | CMT | mNGS | intime_sofa | Ngs_sofa | death_icu_28day | death_time | iculos | los |
| --- | --- | --- | --- | --- | --- | --- | --- | --- | --- | --- | --- |
| ID 289 | 52 | female | Streptococcus pneumoniae | Streptococcus pneumoniae、Candida parapsilosis | 无 | 8 | 8 | YES | 12 |  |  |
| ID 769 | 58 | male | Streptococcus pneumoniae | Streptococcus pneumoniae | Streptococcus pneumoniae、Ebv、cmv | 10 | 10 | No | 28 | 9 | 20 |
| ID1211 | 73 | male | Streptococcus pneumoniae | Streptococcus pneumoniae | Streptococcus pneumoniae | 15 | 17 | YES | 2 | 2 | 2 |
| ID2031 | 75 | male | Streptococcus pneumoniae | Streptococcus pneumoniae | Streptococcus pneumoniae | 14 | 14 | No | 28 | 16 | 42 |
